# Understanding Human Microbiota Offers Novel and Promising Therapeutic Options against *Candida* Infections

**DOI:** 10.3390/pathogens10020183

**Published:** 2021-02-09

**Authors:** Saif Hameed, Sandeep Hans, Ross Monasky, Shankar Thangamani, Zeeshan Fatima

**Affiliations:** 1 Amity Institute of Biotechnology, Amity University Haryana, Gurugram, Manesar 122413, India; saifhameed@yahoo.co.in (S.H.); sandeephans12@gmail.com (S.H.); 2 Department of Pathology and Population Medicine, College of Veterinary Medicine, Midwestern University, Glendale, AZ 85308, USA; rmonas@midwestern.edu

**Keywords:** *Candida*, microbiota, oral, nasopharyngeal, gut, gastrointestinal, vaginal, metabolite

## Abstract

Human fungal pathogens particularly of *Candida* species are one of the major causes of hospital acquired infections in immunocompromised patients. The limited arsenal of antifungal drugs to treat *Candida* infections with concomitant evolution of multidrug resistant strains further complicates the management of these infections. Therefore, deployment of novel strategies to surmount the *Candida* infections requires immediate attention. The human body is a dynamic ecosystem having microbiota usually involving symbionts that benefit from the host, but in turn may act as commensal organisms or affect positively (mutualism) or negatively (pathogenic) the physiology and nourishment of the host. The composition of human microbiota has garnered a lot of recent attention, and despite the common occurrence of *Candida* spp. within the microbiota, there is still an incomplete picture of relationships between *Candida* spp. and other microorganism, as well as how such associations are governed. These relationships could be important to have a more holistic understanding of the human microbiota and its connection to *Candida* infections. Understanding the mechanisms behind commensalism and pathogenesis is vital for the development of efficient therapeutic strategies for these *Candida* infections. The concept of host-microbiota crosstalk plays critical roles in human health and microbiota dysbiosis and is responsible for various pathologies. Through this review, we attempted to analyze the types of human microbiota and provide an update on the current understanding in the context of health and *Candida* infections. The information in this article will help as a resource for development of targeted microbial therapies such as pre-/pro-biotics and microbiota transplant that has gained advantage in recent times over antibiotics and established as novel therapeutic strategy.

## 1. Introduction

Invasive fungal infections may be life-threatening due to occurrence of diabetes and immuno-compromization caused by AIDS, cancer, organ transplants, corticosteroids, post-surgical care, broad-spectrum antibiotic use, etc. [1]. Localized fungal infections that are usually apparent can become systemic, causing deep seated infections as the disease occurrence persists. Despite significant improvements in antifungal drug development, there are still a limited number of available antifungal drugs that are effective. Among infections caused by these opportunistic fungal pathogens, *Candida* species are responsible for the most threatening, invasive mycotic infection and candidiasis. *Candida albicans* is responsible for over half the cases of candidiasis in humans. Over the past two decades, many non-*Candida albicans Candida* (NCAC) species have also emerged as significant pathogens of clinical importance [2,3]. These species are a highly diverse group of organisms and are fundamentally different from each other, including *C. albicans,* at the biological level [4]. This diversity in the range of *Candida* species now associated with human infections has provided new challenges in the diagnosis and treatment of candidiasis and in the study of their virulence and biology. Additionally, species previously considered as nonpathogenic, *C. glabrata*, *C. krusei,* and *C. lusitaniae* have also emerged as pathogens [5,6]. A number of NCAC species are now recognized to exhibit intrinsic resistance to antifungals. Because of their evolutionary similarity to their human hosts, fungal pathogens create additional therapeutic challenges when designing interventions. This similarity restricts the potential drug targets available to selectively kill the pathogen without affecting the host [7]. As a result, there is urgency for deployment of novel approaches to surmount the *Candida* infections.

The pathogenesis of *Candida* spp. is governed by several factors that include yeast to hyphal and phenotypic switching, biofilm formation, adherence and invasion, metabolic flexibility, genome plasticity, pH stress and limiting micronutrients, escape from phagocytosis, host immune evasion, resistance to antifungal agents, and interaction with resident microbiota [8]. The human microbiota is microbial communities that reside inside or on the external surface of the human body, and their genetic makeup is known as the human microbiome [9]. Each microbiota has a unique, synergistic relationship of microbes with the host that acts as the first line of defense against the invading opportunistic pathogens. This mutually beneficial state is known as eubiosis, contrary to disrupted symbiotic balance, occurring by outweighing of opportunistic pathogens, referred to as dysbiosis. Thus, other microorganisms residing in the human body play a major role in human health and diseases progression. Little is known about how microbes interact with each other and influence the host immune system. Understanding the microbiota thus represents a critical aspect in gaining insight into disease progression and prevention. Herein, we reviewed the recent information of the various microbiota present in humans and their influence on the opportunistic *Candida* spp. This will help establishing a resource with information for identifying distinct microbiota profiles associated with *Candida* infections.

## 2. Types of Microbiota

### 2.1. Oral Microbiota

The oral cavity is the first digestive organ that metabolizes starches and dietary lipids, two significant vitality sources for physiology and microbial development. The oral microbiota is a unique and significant part of the human microbiota, and comprises the microorganisms existing in the human oral cavity. The oral cavity is understood to have the second most complex microbiota inside the human body, comprising extremely diverse organisms from all domains of life. Among nearly 700 species found in the oral cavity, about 54% have been cultivated and named, 14% are cultivated but unnamed, and 32% are recognized simply as unrefined phylotypes (from the Human Oral Microbiome Database) [10].

#### 2.1.1. Microbial Niches of Oral Cavity

The oral microbiota plays a significant role in maintenance of mouth homeostasis. Every person’s oral microbiota comprises a diverse and varied group of microbes. Due to its position, microbial growth in the oral cavity is affected by several variables including: personal hygiene, nutrition, and smoking in some cases along with host driven genetic factors that may have additional effects on the oral microbes [11]. The humid surroundings of the oral cavity are maintained at a relatively consistent temperature (34–36 °C), although the fluctuating pH levels, and the factors listed above also impact the microbiome variability.

In the oral microbiota, bacteria are the main oral microorganisms. The oral bacterial community is comprised primarily of these six major phyla, *Firmicutes*, *Bacteroidetes*, *Proteobacteria*, *Actinobacteria*, *Spirochaetes* and *Fusobacteria*, with around 94% of the taxa detected [12,13,14] (Table 1). The other phyla, *Saccharibacteria*, *Synergistetes*, *Gracilibacteria*, *Chlamydia*, *Chloroflexi*, *Tenericutes*, and *Chlorobi*, comprise the remaining 6% of the taxa. The human microbiota diversity is not restricted to bacteria but also extends to fungi as well. Fungi are found in the oral microbiota as both opportunistic pathogens and as members of healthy oral microbiota. Many studies reported that 101 fungal species are existent in healthy people in which *Candida* species are the most common, followed by *Cladosporium*, *Aureobasidium*, *Saccharomyces*, *Aspergillus*, *Fusarium*, and *Cryptococcus* [15]. The most frequent fungal species is *C. albicans* followed by *Candida parapsilosis, Candida tropicalis, Candida khmerensis,* and *Candida metapsilosis* [16]. Archaea consist as a minor part and only a few species are found in the oral microbiota like *Thermoplasmatales*, *Methanobrevibacter*, *Methanobacterium*, *Methanosarcina*, and *Methanosphaera* [17,18]. Of note, some viruses found in the mouth such as Herpes virus cause significant disease of the mouth and mucocutaneous surfaces and can cause recurrent, chronic lesions on the face and lips [19].

#### 2.1.2. *Candida* and Bacteria Interaction in Oral Cavity

*C. albicans* is the fungus commonly found in the oral cavity through any dysbiotic illness, while the predominant bacterial species is *S. mutans*. The proximity of these microbes in youth caries and dental plaques [31] indicates that these organisms may have interactions which impact the sickness. Some in vitro studies reported *C. albicans* initiated articulation of the *S. mutans* destructiveness quality of glucosyltransferase B (GtfB) [14]. Remarkably, GtfB can likewise adhere to the outside of *C. albicans*, promoting adherence to dental surfaces and prompting the development of blended biofilms [32]. RNA-Seq analysis performed on co-cultures revealed that the closeness of *C. albicans* impacts carbohydrate digestion pathways in *S. mutans* [33]. Additionally, the accumulation of *Lactobacillus salivarius* with many species forms biofilms but can obstruct development of *S. mutans* and *C. albicans* biofilms in vitro [34]. Metabolic investigations of both species consequently found intermediate increases in abundance of formate and farnesol. The most abundant molecule detected, farnesol, improves *S. mutans* cell development and micro colony growth in biofilms comprised of both *C. albicans* and *S. mutans* [35]. Further studies convey proof for *S. mutans* reducing the pathogenicity of *C. albicans*. Infusion of *S. mutans* cells consumed by *C. albicans*-infected *Galleria mellonella* larvae enhances endurance of the creatures [36]. Furthermore, AgI/II family polypeptides in *S. mutans* have connections with *C. albicans* and help establishment of two microorganisms in a *D. melanogaster* in vivo model [37].

The streptococci mitis group has been well-known to interact with *C. albicans*. The mitis group are typical oral commensal bacteria, which includes 13 types of streptococci with four species, namely, *S. gordonii, S. mitis, S. oralis, and S. sanguinis*, prevailing in their capacity to communicate directly with *C. albicans* [38]. Additionally, a previous murine model study showed better establishment of *S. oralis* in the oral microbiota in the occurrence of *C. albicans.* Moreover, co-infection supported profound organ propagation of *C. albicans* [39]. In addition, discharge of *S. gordonii* QS molecule autoinducer-2 (AI-2) induces higher hyphae expansion through co-incubations [40]. In a murine co-infection model, there were enhanced levels of proteolytic host protein µ-calpain targeting E-cadherin leading to activation of systemic invasion of *C. albicans*. *S. sanguinis* showed synergistic effects with *C. albicans* through biofilm formation [41]. *S. sanguinis* produces a bacteriocin which showed antibacterial activity against *P. gingivalis* and *P. intermedia* and affected filamentation of *Candida* spp. [42].

Besides streptococci, numerous other oral microorganisms also interact with *C. albicans*. For instance, the periodontal pathogens *Aggregatibacter actinomycetemcomitans* and *Fusobacterium nucleatum* impede propagation of *C. albicans* by elimination of quorum sensing molecule AI-2 [43]. Co-cultivation of *C. albicans* and anaerobic *Porphyromonas gingivalis* in standard oxygen conditions enhances growth of *P. gingivalis* in the presence of *C. albicans* biofilm, which suggests protection of *P. gingivalis* under aerobic conditions [44]. Moreover, an in vitro experiment validates *P. gingivalis* being able to interrupt oral epithelium cell movement while interrelating with another *Candida* spp. such as *C. kefyr* and *C. glabrata* [45]. A study displayed that *Actinomyces* species like *A. viscosus*, *A. naeslundii*, and *A. odontolyticus* could constrain the growth of *C. albicans*. *C. albicans* adherence to oral epithelial cells was reduced and production of phospholipase C was improved in the presence of supernatants from *Actinomyces* spp. [29]. *Rothia dentocariosa* is developing as an opportunistic bacterium and usually is collected with *C. albicans* from failed silicone voice prostheses in subjects who endured laryngectomy [46]. *S. aureus* is also normally found in healthy oral microbiota, [47] along with oral diseases which generally are related to the incidence of *C. albicans*, like denture stomatitis [48] and angular cheilitis [49].

#### 2.1.3. Oral Dysbiosis

*C. albicans* colonization in mucosal sites is restricted by commensal bacterial communities. However, under immunosuppressed conditions when the microbial homeostasis is changed certain bacterial species form mutualistic connections with *C. albicans* [50,51]. Immunocompromised conditions led to dysbiosis leading to mucosal damage. A change towards dysbiosis is usually led by dietary changes or antibiotics reducing diversity. Changes in the homeostatic balance lead to bacteria-mediated diseases such as dental caries, endodontic infections, gingivitis, and periodontitis [52,53]. Oral candidiasis leads to damage to the oral mucosal surface and is caused when the superficial tissue layers are colonized, overgrown, and invaded by *Candida* species. One cause of oral *Candida* overgrowth is dysbiosis due to immunosuppression, for instance, HIV infection [54].

### 2.2. Nasopharyngeal Microbiota

The nose and nasopharynx are vital niches colonized by commensal and potential pathogenic species which together comprise the nasopharyngeal microbiota. In humans and mammals, the nasal cavity is well identified as an essential site for colonization by diverse bacterial and fungal species. The nasopharynx is the dominant microbiota present in other regions of the upper respiratory tract, and the nasopharyngeal (NP) microbiota arises from early colonization. In humans, although *Candida* spp. are not considered as significant constituents of the upper respiratory tract microbiota, their existence may be relevant to immune responses in this region.

#### 2.2.1. Microbial Niches of Nasopharyngeal Microbiota

NP microbiota profiles are not stable during early development, and the microbiota transforms as development occurs. For example, in infants, NP microbiota are characterized by majorly the phyla *Proteobacteria, Firmicutes, Bacteroidetes, Actinobacteria,* and *Fusobacteria*, with the most prominent genera being *Moraxella, Haemophilus, Streptococcus, Dolosigranulum, Corynebacterium,* and *Neisseria* [55] (Table 1). In the case of detected genera in adults, while *Staphylococcus, Haemophilus,* and *Streptococcus* are extant, also present are *Sphingobacterium, Prevotella, Bifidobacterium, Rothia, and Propionibacterium,* but neither *Moraxella* nor *Corynebacterium* [20]. In the case of fungi, among the 25 species of yeasts in the NP microbiota that are considered as emerging pathogens, 20 are *Candida* spp. [21].

#### 2.2.2. *Candida* and Bacteria Interaction in Nasopharyngeal Microbiota

The NP microbiota has substantial microbial interactions in the form of synergism and antagonism. During *S. aureus* colonization, *H. influenzae* and *M. catarrhalis* display a strong negative impact on *S. aureus* colonization compared to *S. pneumoniae* [56]. Strong positive relations in colonization amongst *S. pneumoniae*, *M. catarrhalis,* and *H. influenzae* have also been documented [57,58]. The synergistic association is that colonization of *S. pneumoniae* and *H. influenzae* rises during co-culture [59]. *M. catarrhalis* shows a strong interaction with *H. influenzae,* by avoiding complement-mediated killing through accompaniment inhibition of C3. A study revealed that commensal microbes understood to have little pathogenic potential also communicate in this environment [60]. For instance, *Corynebacterium* and *Dolosigranulum* were earlier believed to be factors in reducing *S. aureus* abundance [61]; however, not all *Corynebacterium* spp. were shown to inhibit *S. aureus* [62]. *Pneumococcus* has displayed greater biofilm density when forming multi-species biofilms with *H. influenza*. Regarding the role of fungi in the NP microbiota, it includes some specific species that help in interaction with other species of bacteria and fungi. *Aspergillus, Candida,* and *Cryptococcus* are found in respiratory samples from persons with multiple risk factors such as immunodeficiencies [63], and it has been established that fungal colonization can be related with asthma and chronic respiratory disease [64]. Furthermore, in a rat model, *C. albicans* damages macrophage-mediated clearance of *Pseudomonas aeruginosa* during pneumonia [65], and in biofilm models, it also influences infection and mucosal inflammatory response towards Streptococcal disease [66]. 

#### 2.2.3. Nasopharyngeal Dysbiosis

NP dysbiosis alters the commensal microbiota, disrupting homeostasis, and this dysbiosis influences mucosal and systemic immunity by creating inflammatory responses within the host [67]. Direct colonization of the upper respiratory tract in elderly populations is low for pathogens such as *Streptococcus pneumonia*, a major cause of pneumonia and more invasive disease [68]. Additionally, murine models show the composition of the NP microbiota changes during *S. pneumoniae* colonization in which the elderly mice were not able to clear *S. pneumoniae* as successfully as the young mice [69]. However, other studies suggest that the NP microbiota is the important factor in this colonization, as the most assorted adult microbiotas are also the lowest expected to find *S. pneumoniae* [70]. The NP commensal *Haemophilus influenzae* is among six capsulated varieties and was a major contributor in life-threatening infections like meningitis and septicemia. Moreover, non-typable *Haemophilus influenza* (NTHi) is also responsible for acute, mucosal or chronic infection. Additionally, the impact of *M. catarrhalis* can be seen prominently in acute mucosal infections and in chronic obstructive pulmonary disease (COPD) [71]. Additional severe diseases are also recognized as a consequence of dysbiosis and comprise bacteremia, sepsis, mastoiditis, septic arthritis, and endocarditis [72,73,74].

### 2.3. Gut and Gastrointestinal Microbiota

The human gut and the gastrointestinal microbiota are a composite, populated by diverse species of bacteria, fungi, and archaea. The gut is a niche for billions of microorganisms that help metabolism and digestion in their symbiotic relationship with the host. Many reports have demonstrated that gut microbiomes have lesser fungal burden contrary to bacterial communities. Fungal composition is about 0.1% of the gastrointestinal tract microorganisms, and there is an established relationship of antagonism and synergy between fungi, bacteria, and viruses [75]. In the adult individual, the gut microbiome has approximately 10^14^ bacteria. The microbes residing in the gut are discrete from the microbes linked to the microbiota of other parts of the host [76]. Many large-scale projects such as Metagenomics of the Human Intestinal Tract (MetaHIT) and National Institutes of Health (NIH) Human Microbiome Project examining the gut microbiota have attracted considerable interest due to the abundance and diversity of bacterial microbiota existing in the gut [22,76]. 

#### 2.3.1. Microbial Niches of Gut and Gastrointestinal Microbiota

The majority of bacteria in the gut of healthy persons are associated with the phyla, *Bacteroidetes,* and *Firmicutes* (collected 70–90%) and to a lesser extent, *Actinobacteria* and *Proteobacteria*. These phyla represent the core microbiota of the human gut [23,24]. Inside the gut ecosystem several factors which include oxygen levels, changes in pH, or macro- and micronutrients variation, affect the variety and abundance of bacterial and fungal species. The gut microbiota helps in the absorption of nutrients and water, and a semipermeable barrier is assembled essentially by enterocytes (intestinal epithelial cells). Ascomycota and Basidiomycota are the common taxa identified in the gut mycobiota from healthy individuals, with leading genera: *Saccharomyces, Candida, Malassezia,* and *Cladosporium* [22,25]. In the gastrointestinal (GI) tract of humans, bacterial genera that are most usually found include *Clostridium*, *Streptococcus*, and *Bacteroides* (Table 1). The colon is the leading site for the bacterial fermentation of non-digestible food such as fibers, and it comprises about 70% of all host bacteria. The studies reported that fungi belong to the phyla Ascomycota, Basidiomycota, and Chytridiomycota are the most common [77]. More consistently found fungi are also restricted, with the genera *Candida* and *Saccharomyces* being the prominent ones [26]. The most frequently described genus of Archaea that is found in the GI tract is *Methanobrevibacter* while others are also been detected including *Methanosphaera*, *Nitrososphaera*, *Thermogynomonas*, *Thermoplasma,* and *Methanomethylophilus alvus*. The solitary diet factor inducing fungal colonization and growth in the GI tract in the presence of *Candida* spp. are mostly linked with dietary carbohydrates, and not with dietary amino acids, proteins, and fatty acids.

#### 2.3.2. *Candida* and Bacteria Interaction in Gut and Gastrointestinal Microbiota

Generally, most animals, including humans, with intact, healthy microbiota are unaffected by pathogenic fungi, viz. *C. albicans* colonization [78]. *C. albicans* is commensal in the human gut, providing an insight into the connections between the fungal and bacterial species in the gut. The occurrence of *Firmicutes* and *Bacteroides* appears significant to sustain *C. albicans* growth persistence in a mouse model of infection [79]. Fungal-bacterial community interaction was shown in mice treated with dextran sulphate sodium (DSS) to induce colitis. In this model, DSS-treatment inhibited growth of *C. glabrata*, which in turn reduces infection. It directed increased growth of *E. coli, E. faecalis,* and *Bacteroides vulgatus,* although *Lactobacillus johnsonii*, *Bacteroides thetaiotaomicron*, and *Bifidobacterium* diminished [80] (Table 1). The strain *Saccharomyces cerevisiae boulardii* is used as a defensive measure against *Clostridium difficile* infection. The colonization of *C. albicans* can change the total benefits of commensal enteric bacteria. [81]. *S. cerevisiae* diminished bacterial growth, reduced colonization, and inverted the attachment of enterotoxigenic *E. coli* (ETEC) to the host [82]. *Salmonella* Typhimurium inhibits *C. albicans* hyphal formation, biofilm, etc. When a SopB (TTSS) knockout mutant is used, this inhibition is lower [83]. *C. albicans* hyphal growth is regulated by novel role of TOR1 governing the activities of adhesion genes, hinting at the mammalian target of rapamycin (mTOR) signaling pathway [84]. On the other hand, as demonstrated in a peritonitis murine model, fungal-bacterial communication can show synergistic effects on the host through proinflammatory cytokines secretion, with the consequence of cell destruction on the host [85]. Some communities of bacteria and fungi interact with each other and help to colonize the gut and oral cavity. This biofilm comprised of multiple species can provide additional defense against antimicrobial agents and provide host immune evasion [86]. *C. albicans* infection into mice chemotherapy model induces important changes in gut microbiota diversity. *Enterococcus* spp., specifically *E. faecalis,* were found to promote *Lactobacillus* growth upon subsequent infection of *C. albicans* into the gut of chemotherapy model of mice [87]. In a *Caenorhabditis elegans* model of infection [88] *E. faecalis* inhibited *C. albicans* yeast to hyphal transition by release of proteases involved with quorum sensing. In a mouse model, *Lactobacillus* and *C. albicans* may compete with one another [89]. The *E. faecalis* bacteriocin EntV was recognized as the important inhibitor of hyphal development [90]. *C. albicans* and *E. faecalis* are often identified together in nosocomial infections which suggest that *E. faecalis* could utilize alternate mechanisms for accommodating growth with *C. albicans.* Interestingly, fungi have a role in the gut-brain axis. Fungi are prominent in the bidirectional connections amongst brain and gut through neuro-immuno-endocrine mediators, which is equivalent to microbiome-gut-brain axis [91]. In an in vivo model, a synergistic effect was observed between *C. albicans* and *Streptococci. Streptococci* enhances invasion by *C. albicans*, while *C. albicans* improves *Streptococcus* spp. biofilm development in the superior GI mucosa [92]. Similar interactions have been studied between *C. albicans, Lactobacillus spp., H. pylori,* and *Escherichia coli* in the GI tract [93]. For instance, the co-cultures of *C. albicans* with *H. pylori* increase the disease progression of GI ulcers. Additionally, *Lactobacillus* spp. inhibit the growth and pathogenesis of *C. albicans* through hydrogen peroxide production, although this inhibition was not sufficient to eliminate *C. albicans* [94]. Similarly, diverse *Lactobacillus* species have changed their capabilities in obstructing the growth of *C. albicans* in the GI tract. Moreover, the variation seen in fungal virulence may be influenced by the impact of *Lactobacillus* spp. on the host immune response [95]. In the GI tract disruptions of the bacterial community tend to promote *C. albicans* colonization, and this result indicates that the normal homeostasis of the bacterial species in the GI microbiota inhibits fungal expansion and invasion [96]. *Saccharomyces boulardii* is an exogenous fungal probiotic, extensively used as a probiotic in scientific practice ever since the 1950s in Europe [97].

#### 2.3.3. Gut and Gastrointestinal Dysbiosis

Fungal dysbiosis in the gut contributes to the establishment and pathogenesis of several human diseases. Some important factors contribute toward fungal growth progression, which include many gut-related diseases such as inflammatory bowel diseases, Crohn’s disease, ulcerative colitis, and gut inflammation [98]. In mice treated with antifungal drugs to induce dysbiosis in the gut, the level of opportunistic fungi like *Aspergillus amstelodami, Epicoccum nigrum*, and *Wallemia sebi* increases [27]. It may be possible to associate gut microbiota dysbiosis with digestive tract diseases/diet linked diseases (obesity, inflammatory bowel disease, enterocolitis, diabetes, etc.). Gut dysbiosis is also involved in diseases outside the intestine such as muscular dystrophy, mental disorders, cancers, vaginosis, etc. [99]. Furthermore, gut microbiota dysbiosis have been associated with ocular diseases (which are as remote as the brain), Uveitis, age-related macular degeneration, Sjogrens syndrome-associated dry eye and Keratitis. Fungal dysbiosis influenced by anti-fungal action affected a selection of fewer, non-*Candida* fungi that caused deterioration of DSS-induced colitis and exacerbated allergic airway disease [100,101]. Broad-spectrum antibiotic implementation is a unique threat factor for systemic, disseminated candidiasis originating in the gut microbiota as antibiotic treatment diminishes bacteria that normally limit *Candida* growth [102]. GI dysfunction occurs mainly due to motility and absorption disturbances, mucosal disintegrity, alterations in the microbiome, enhanced intra-abdominal pressure and altered infections of the GI tract. Many reports demonstrated the dysbiosis between the bacterial communities in the GI tract including inflammatory bowel diseases, transplantation, gastric ulcer and gastritis, blood diseases, HIV infection, and other immunodeficiencies and antibiotic associated diarrhea. *C. albicans* was recovered in the stools of 63% healthy individuals, 70% of inpatients, and 91% of patients with inflammatory bowel disease [103,104]. The colonization of *Candida* in the GI tract has also been associated with other conditions like diabetes [105]. Some specific opportunistic pathogens such as *Aspergillus flavus, Basidibolus ranarum, Cryptococcus noeformans, Exophilia dermatidis, H. Capsulatum, Paracoccidioides brasiliensis*, and *Penicilliummarneffei* were found in immunocompromised/immunosuppressed groups and in antibiotic associated diarrhea [106,107,108].

#### 2.3.4. *Candida* and Metabolite Interaction in the Gastrointestinal Tract

In addition to the wide range of microbiome interactions taking place within the GI tract, metabolites present in the GI tract have a significant role in the colonization and pathogenesis of *Candida albicans* in the host. A recent metabolomics study conducted by our group using a mouse model of *C. albicans* infection identified significant changes in the gut metabolome as a result of antibiotic treatment [109]. Among the metabolites that were differentially regulated, three prominent groups were identified: carbohydrates and sugar alcohols, bile acids, and short-chain fatty acids (SCFA). Further investigation into each group and their interactions with *C. albicans* revealed a wide range of effects suggesting prominent roles for specific metabolites in promoting or inhibiting *C. albicans* colonization and pathogenesis.

Primary bile acids, specifically taurocholic acid (TCA) and taurochenodeoxycholic acid (TCDCA) were found to be upregulated in this metabolomics study, and in vitro incubation of *C. albicans* with these metabolites revealed a significant increase in fungal growth and hyphal morphogenesis [109,110,111]. Further, carbohydrates and sugar alcohols significantly promoted the fungal growth and morphogenesis in vitro. In contrast, the levels of secondary bile acids in the metabolomics study were decreased, and multiple studies have identified secondary bile acid such as deoxycholic acid (DCA), hyodeoxycholic acid (HDCA), ursodeoxycholic acid (UDCA), and lithocholic acid (LCA) as inhibitory for in vitro *C. albicans* growth as well as inhibiting hyphal formation in vitro and ex vivo [109,111,112]. The secondary bile acids LCA and DCA were also found to inhibit biofilm formation and pre-treatment of HCT116 cells with these bile acids reduced *C. albicans* attachment [112]. SCFA levels are also decreased during antibiotic treatment, and in vitro growth of *C. albicans* is inhibited by SCFAs such as acetic acid, butyric acid, and propionic acid. This study also demonstrated that these SCFAs reduce in vitro hyphal formation and reduce metabolic activity of *C. albicans* biofilms [113]. Taken together, emerging evidence indicate that gut metabolites differentially control the fungal growth and hyphal morphogenesis. Additional investigation is needed to dissect how these metabolites regulate fungal colonization and pathogenesis, to better understand fungal commensalism and pathogenicity and to develop novel therapeutic approaches.

### 2.4. Vaginal Mycobiota and Dysbiosis

The female vaginal tract of healthy women of childbearing age comprises mainly of *Lactobacillus* which is the first line of defense against pathogens. Additionally, there are fungal communities that exist within the interior female reproductive tract. The three fungal phyla identified in vaginal sections are Ascomycota (in which *Candida* is the main genera), Basidiomycota, and Oomycota [114] (Table 1). Many studies showed that the composition of the fungal mycobiota of the vagina has great significance because its disturbance is the leading cause of many vaginal infections. These infections comprise bacterial vaginosis, vulvovaginal candidiasis, aerobic vaginitis, chlamydia infection, gonorrhea, and other sexually transmitted diseases including human immunodeficiency virus (HIV). Bacterial vaginosis and vulvovaginal candidiasis are the most prominent infections disturbing the female reproductive tract [115,116]. Bacterial vaginosis is of particular concern for pregnant women as it increases risk for preterm delivery, maternal infectious morbidity and a resilient hazard for late miscarriage [117].

#### 2.4.1. Microbial Niches of Vaginal Mycobiota

The organization of the vaginal bacterial and fungal species of fit women is complex due to variations associated with menstruation, pregnancy, and health prominence. The vaginal niche of a healthy women is distinctly acidic (pH of 4.5 or less), which is mainly due to the occurrence of lactic acid producing bacteria that survive in the anaerobic conditions [118]. The hormone-dependent glycogen produced by human vaginal epithelial cells and other nutrients including lactate contribute to fungal colonization. *Candida* is extremely flexible regarding shifts in the nutrients available in the vagina. The native vaginal microbiota mainly is a consolidation of genera *Lactobacillus, Gardnerella, Atopobium, Prevotella, Streptococcus, Ureaplasma, Escherichia, Mycoplasma, Staphylococcus Candida, Megasphaera,* and many others [29]. Among these, *Lactobacillus* spp. is the vital member of the vaginal microbiota, which ensures a healthy niche. *Candida* species are normal component of their vaginal microbiota (e.g., *C. tropicalis, C. pseudotropicalis, C. stellatoidea, C. krusei, C. guilliermondii,* and *C. albicans*). 

#### 2.4.2. *Candida* and Bacteria Interaction in Vaginal Microbiota

Lactobacillus in the vaginal microbiota is largely associated with protecting the vaginal mucosa and creating a healthy microenvironment. Decreased *Lactobacillus* spp. is associated with vulnerability to multiple diseases, including bacterial vaginosis, urinary tract infections, vulvovaginal candidiasis, and other urogenital infections. Lactobacilli are believed to inhibit *C. albicans* by numerous mechanisms. Lactobacilli compete with *C. albicans* for limited nutrients and adherence sites, reducing growth, along with preventing hyphal development [119]. *C. albicans* attachment to vaginal epithelial is diminished during co-colonization with multiple *Lactobacillus* spp. [120]. A bacteriocin-like peptide produced by multiple *L. crispatus* and *L. jensenii* strains influence pseudohyphae formation and inhibited growth of *C. albicans* from healthy premenopausal women [121]. *L. crispatus* can reduce *C. albicans* virulence and increase the local immune response by promoting immune modulating cytokines profile, i.e., upregulating IL-2, IL-6, and IL-17 and downregulating IL-8 [122]. Additionally, some studies showed that *C. albicans* is susceptible to lactic acid at low pH. They proposed that lactic acid invades through the fungal plasma membrane at lower pH levels where it separates into protons and lactic acid [123]. The ions acidify the cytosol and restrict the cell metabolism. Moreover, lactobacilli also reduce *C. albicans* virulence factors by the inhibition of hyphae growth [124]. An in vivo study by using the nematode model *Caenorhabditis elegans*, showed that *L. paracasei* repressed *C. albicans* hyphae growth. Similarly, *Galleria mellonella* larvae treated with *L. rhamnosus* enhanced survival after *C. albicans* infection and reduced fungal colony forming units [125]. *Candida*-bacteria connections inside the vagina are likely to occur in the interior context of a polymicrobial biofilm on the epithelial surface. The synergistic association between *C. albicans* and Group B *streptococci* and *Escherichia coli* is harmful for the host. Some studies validated an in vitro model with vaginal epithelial cells where Group B *streptococci* and *C. albicans* synergistically improved their ability to reside with the host cells [126].

## 3. Targeted Microbial Therapy

Microbiota restoration strategies are required for treatment which allow beneficial bacteria to propagate, eliminate colonization by opportunistic pathogens, and enhances resistance to colonization by pathogens. Prebiotics can affect the bacterial population by benefitting the host through specific environmental or direct interactions with pathogenic microbes and immune cell carbohydrate receptors. Neutrophil phagocytosis is initiated through receptors on phagocytes and natural killer cells that bind β-glucans on *Candida* cell walls. Additionally, prebiotics encourage health by supporting the production of metabolites, e.g., folate, indoles, secondary bile acids, and short-chain fatty acids. Prebiotic treatment helps in selective upsurge/drop in specific microbiota bacteria that ultimately can induce health benefits [127]. They modulate the microbiota and exert a downstream effect on host health. Probiotics are getting hold of extensive inclusion as novel prevention strategies or treatments for a wide range of diseases, as validated by many studies. Mechanisms of probiotics comprise restoration of microbial populations and destruction of pathogens, increase of anti-inflammatory cytokines and suppression of proinflammatory cytokines along with improvement of immunity [128]. How probiotics may mediate such beneficial effects, including direct inhibition of pathogens, has been demonstrated by several mechanisms. Postbiotic supplementation derived from *Lactobacillus paracasei* was able to protect *G. mellonella* infected with *C. auris* and immunomodulate indicating a dual role in modulating host immune response [129]. Even meta-analysis is among the first study of such kind which establishes the effect of probiotics on oral candidiasis [130].

## 4. Microbiota Transplant

Fecal microbiota transplantation (FMT) is the transmittance of donor microbiota inoculum such that damaged microbiota is restored. It has been recently successfully applied to ulcerative colitis where the intestinal microbiota of the patients sustains severe damage from multiple rounds of antibiotic treatment. This strategy works on an organ or tissue “transplant” therapeutic model and recognizes microbiota as a complex unit. FMT reduces *Candida* by suppressing pro-inflammatory immune responses during intestinal disease [131]. Another study revealed that FMT is effective in preventing Candida colonization of the GI tract [132]. However, this is a provisional strategy until an actual drug product is approved to fulfill the unmet need.

## 5. Conclusions

There are limited studies unravelling the mystery regarding the composition and diversity of human microbiota and its interrelation with *Candida* infections. The current scenario reveals a need for comparative genomics-based studies on pathogens to enlighten our rudimentary understanding of fungal pathogenesis. Similarly, targeted microbial therapy and microbial transplant are novel methods that are yet to be explored to their potential. In silico metabolic interaction predictions can also lead to identification of microbiome features affecting *Candida* colonization. Comprehending these factors may help in the development of a better perception of how antagonistic and synergetic microbial interactions influence *Candida* infections so that targeted microbial therapy could be employed.

## Figures and Tables

**Table 1:** Diverse microbiota depicting resident phyla along with those associated with *Candida* infections.

Types of Microbiota	Resident Phyla	Phyla Influencing *Candida* Infections	Reference
Oral	*Firmicutes, Bacteroidetes, Proteobacteria, Actinobacteria, Spirochaetes, Fusobacteria, Saccharibacteria, Synergistetes, Gracilibacteria, Chlamydia, Chloroflexi, Tenericutes* and *Chlorobi, Ascomycota, and Basidiomycota*	*Firmicutes (Streptococcus, Lactobacillus),* *Bacteroidetes (Porphyromonas),* *Proteobacteria (Aggregatibacter),* *Actinobacteria (Actinomyces),*	[12,13,14,15]
Nasopharynx	*Proteobacteria, Firmicutes, Bacteroidetes, Actinobacteria and Fusobacteria, Ascomycota,*	*Proteobacteria (Pseudomonas)*	[20,21]
Gut	*Bacteroidetes, Firmicutes, Actinobacteria, Proteobacteria, Ascomycota, and Basidiomycota*	*Firmicutes (Streptococcus, Lactobacillus),* *Bacteroidetes (Bacteroides),* *Proteobacteria (Esherichia, Salmonella)*	[22,23,24,25]
Gastrointestinal	*Bacteroidetes, Firmicutes Ascomycota, Basidiomycota Chytridiomycota* and *Euryarchaeota*	*Firmicutes (Streptococcus, Lactobacillus),* *Proteobacteria (Esherichia, Helicobacter)*	[26,27]
Vaginal	*Firmicutes, Bacteroidetes, Proteobacteria, Actinobacteria, Tenericutes, Ascomycota, Basidiomycota,* and *Oomycota*.	*Firmicutes (Lactobacillus, Streptococcus),* *Proteobacteria (Esherichia)*	[28,29,30]

## Data Availability

All data presented in this study are available in this manuscript.
